# The patient-breast cancer care pathway: how could it be optimized?

**DOI:** 10.1186/s12885-015-1417-4

**Published:** 2015-05-12

**Authors:** Sandrine Baffert, Huong Ly Hoang, Anne Brédart, Bernard Asselain, Séverine Alran, Hélène Berseneff, Cyrille Huchon, Caroline Trichot, Aline Combes, Karine Alves, Martin Koskas, Thuy Nguyen, Aurélie Roulot, Roman Rouzier, Delphine Héquet

**Affiliations:** 1 Department of Health Economy, Institut Curie, 26 rue d’Ulm, 75005 Paris, France; 2 Department of Supportive Care, Institut Curie, 26 rue d’Ulm, 75005 Paris, France; 3 Department of Biostatistics, Institut Curie, 26 rue d’Ulm, 75005 Paris, France; 4 Department of Surgical Oncology, Institut Curie, 26 rue d’Ulm, 75005 Paris, France; 5 Department of Gynecology, René Dubos Hospital, 6, avenue de L’Ile de France, 95303 Pontoise, France; 6 Department of Gynecology, Poissy-St Germain hospital, 10 Rue du Champ Gaillard, 78300 Poissy, France; 7 Department of Gynecology, Antoine Béclère Hospital, 157 rue de la Porte de Trivaux, 92140 Clamart, France; 8 Department of Gynecology, André Mignot Hospital, 50 rue Berthier, 78000, Versailles, France; 9 Department of Gynecology, Argenteuil Hospital, 69 Rue Lt Colonel Prudhon, 95100 Argenteuil, France; 10 Department of Gynecology, Bichat Hospital, 46 rue Henri Huchard, 75018 Paris, France; 11 Department of Gynecology, Louis Mourier Hospital, 178 rue des Renouillers, 92700 Colombes, France; 12 Department of Surgical Oncology, Institut Curie-René Huguenin, 35 rue Dailly, 92210 St Cloud, France; 13 Equipe d’Accueil 7285, Risk and safety in clinical medicine for women and perinatal health, University Versailles-Saint-Quentin, Montigny-le-Bretonneux, France

**Keywords:** Care pathway, Breast cancer, Cost analysis, Bundled payment

## Abstract

**Background:**

A care pathway is defined as patient-focused global care that addresses temporal (effective and coordinated management throughout the illness) and spatial issues (treatment is provided near the health territory in or around the patient’s home). Heterogeneity of the care pathways in breast cancer (BC) is presumed but not well evaluated. The OPTISOINS01 study aims to assess every aspect of the care pathway for early BC patients using a temporal and spatial scope.

**Methods/Design:**

An observational, prospective, multicenter study in a regional health territory (Ile-de-France, France) in different types of structures: university or local hospitals and comprehensive cancer centers. We will include and follow during 1 year 1,000 patients. The study consists of 3 work-packages:

- Cost of pathway

The aim of this WP is to calculate the overall costs of the early BC pathway at 1 year from different perspectives (society, health insurance and patient) using a cost-of-illness analysis. Using a bottom-up method, we will assess direct costs, including medical direct costs and nonmedical direct costs (transportation, home modifications, home care services, and social services), and indirect costs (loss of production).

- Patient satisfaction and work reintegration

Three questionnaires will assess the patients’ satisfaction and possible return to work: the occupational questionnaire for employed women; the questionnaire on the need for supportive care, SCNS-SF34 (‘breast cancer’ module, SCNS-BR8); and the OUTPASSAT-35 questionnaire.

- Quality, coordination and access to innovation

Quality will be evaluated based on visits and treatment within a set period, whether the setting offers a multidisciplinary consultative framework, the management by nurse coordinators, the use of a personalized care plan, the provision of information via documents about treatments and the provision of supportive care.

The coordination between structures and caregivers will be evaluated at several levels. Day surgery, home hospitalization and one-stop breast clinic visits will be recorded to assess the patient’s access to innovation.

**Discussion:**

The assessment of care pathways encourages the implementation of new payment models. Our approach could help health care professionals and policymakers to establish other cost-of-illness studies and plan the allocation of resources on a patient basis rather than a visit basis.

## Background

Health care systems have evolved into care pathway models in response to chronic diseases and the expenditures related to them. Disease management tends to be multidisciplinary and transversal, reducing the historic place of the hospital in favor of other long-term supports. The shortcomings of health care for chronic disease are shared issues for a number of countries. Cancer has become a chronic disease as a result of improved treatment. Breast cancer (BC) is a good example of a chronic cancer. Incidence, therapeutics, practices and costs of BC can vary substantially in a given area. There are 1.15 million incident cases of breast cancer per year worldwide [1]. The disease results in high costs in terms of care, out-of-pocket expenses and losses of productivity; the cost of breast cancer was 126 billion € in 2009 in Europe, corresponding to 12 % of all cancer-related costs [2]. Moreover, the heterogeneity of BC-related care pathways and structures at the regional level are presumed but not well evaluated.

Some international health policies aim to take a more global view of chronic disease. In France, a 2004 law named the referring physician as the organizer of the patient care pathway [3]. In 2009, the HPST law assigned a central role to the regional health agency (ARS) to improve the continuity of care in a given health territory in France [4]. A care pathway is defined by the ARS as patient-focused global care that takes a territorial approach. The concept includes education, prevention, diagnosis, treatment, rehabilitation, surveillance and social care. The care pathway addresses temporal (effective and coordinated management throughout the illness) and spatial issues treatment provided in or near the patient’s home health territory). The aim is to provide well-defined practices at well-defined times and places. Care pathways are also presumed to favor equity by facilitating access to care and to improve efficiency by reducing the inappropriate use of health resources.

National initiatives and incentives have enhanced planned care. The chronic care model [5, 6], the use of patient-centered medical homes (PCMH) or the facilitation of practice in primary care are being actively promoted and tested in the United States along with bundled payment for some surgical treatments [7]. However, all of these processes focus on small portions of the care pathway. Multidimensional evaluations of the BC pathway have not yet been described.

Our study, OPTISOINS01, aims to assess every aspect of the care pathway for early BC patients from a chronological and spatial scope. It will describe the stages of the care pathway, including innovative organization. In this paper, we will describe the objectives and the design of the study.

## Methods

OPTISOINS01 is an observational, prospective, multicenter study conducted with patients from a determined regional health territory. This study was approved by the French National ethics committee (CCTIRS Authorization n°14.602 and CNIL DR-2014-167) and covers research at all participating hospitals.

### Setting and population

The three departments of interest in this study (Hauts-de-Seine, Yvelines, and Val d’Oise) cover 35 % of the population of the Ile-de-France region (total population: 11.9 million). With 2.17 million women living in this area, 61 % of whom are older than 45 years, the incidence and mortality of BC in this area are higher than the national rates. This territory was chosen for its heterogeneity in terms of the health care services provided and for its variation in professional densities and facilities, which is linked to the disparate urbanization and population incomes throughout the territory. Eight nonprofit hospitals participate in the study: three university hospitals (Antoine Béclère hospital, Clamart, France; Bichat-Beaujon hospital, Clichy and Paris, France; Louis Mourier hospital, Colombes, France), four local hospitals (André Mignot, Versailles, France; René Dubos, Pontoise, France; Poissy-St Germain hospital, Poissy, France; Victor Dupuy hospital, Argenteuil, France) and one comprehensive cancer center (Curie Institute, Paris and St Cloud, France). These are the hospitals that treat the most BC patients in the Yvelines, Hauts-de-Seine and Val d’Oise departments. Each hospital will include between 30 and 450 patients over 4–6-month period, depending on the yearly number of BC patients treated. A total of 1,000 patients will be included; approximately 150 will undergo outpatient surgery, and 200 will have home hospitalizations. The inclusion and exclusion criteria are presented in Table 1.

**Table 1:** Patient selection

Inclusion criteria
- Histologically confirmed, previously untreated, operable breast cancer
- Residence in the Yvelines, Hauts-de-Seine or Val d’Oise departments
- Age ≥18 years
- Sex: female
Exclusion criteria
- Previous history of breast cancer
- Metastatic, locally advanced, or inflammatory breast cancer, as defined by the AJCC (7th Edition).
- Unstable over the following 12 months

### Procedures

**Fig. 1:**
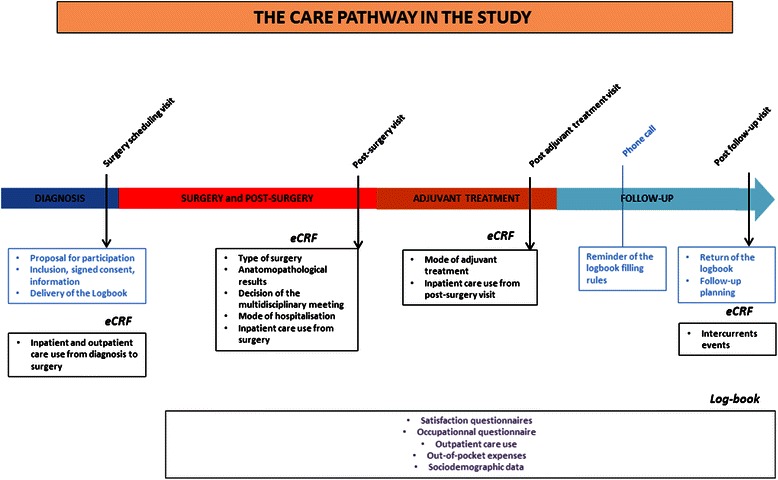
OPTISOIN study design

Patients will be approached for the study during their surgery programming visit. The patients will be followed for 1 year, as described in Fig. 1.

### Objectives and endpoints

This study aims to identify the main care pathway after 1 year of early BC and to evaluate costs from different perspectives. The secondary objectives are to assess patient satisfaction, needs for supportive care, and work reintegration and to evaluate interactions between health care providers in and out of the hospital. In light of these objectives, the study consists of three work packages (WPs).

#### WP1: cost of pathway

The aim of this WP is to calculate the overall costs of the early BC pathway at 1 year from different perspectives (society, health insurance and patient) using a cost-of-illness analysis. Using a bottom-up method, we will assess direct costs, including medical direct costs and nonmedical direct costs (transportation, home modifications, home care services, and social services), and indirect costs (loss of productivity). Productivity losses will be measured in terms of lost wages using a human capital approach (HCA). From a societal perspective, the HCA estimates an individual’s contribution to society by applying labor force earnings as a measure of productivity. We will evaluate the out-of-pocket health expenses for BC. These include the costs associated with the use of health care services, alternative therapies, dietary supplements, specific cosmetic products, capillary prosthesis, clothes, domestic help and travel expenses. The protocol includes different data sources. Each patient will use a logbook to provide information about her socio-demographic status, her type of health insurance, her outpatient consumptions, her out-of-pocket expenses and her modes of work reintegration. The patient will update the logbook monthly from the post-surgical visit until the 1-year follow-up.

#### WP2: patient satisfaction and work reintegration

Three questionnaires will assess the patients’ satisfaction and possible return to work.

- The occupational questionnaire for employed women includes the following items: dates of work and absence from work during treatments, work arrangements, on-shift status (e.g., the recognition of disability at work, applications for disability allowance, retirement, layoff) and the perceived quality of reintegration (e.g., relationships with colleagues and line management).
- The supportive care need survey SCNS-SF34 [8] and its ‘breast cancer’ module, SCNS-BR8 [9] includes six care need subscales: “Physical and Daily Living”, “Psychological”, “Health System and Information”, “Patient Care and Support”, “Sexuality” and “Breast Cancer Specific Needs”. Response scores are standardized on a scale ranging from 0 to 100 with higher scores indicating higher needs. Adequate internal consistency (Cronbach’s α) estimates ranged from 0.80–0.93 in a French validation [10].
- The OUTPASSAT-35 questionnaire [11] is adapted from the EORTC IN-PATSAT32 questionnaire [12] and has 35 items. The first two parts address physician and nurse care in terms of the professionals’ technical skills, relational quality, information provided and availability. The third part broaches service and organization in terms of information sharing among care providers, the identification of a referring physician, information provided by other staff members, wait times, and the physical environment of the hospital. Patients respond to the items using a 5-point scale. Summing the patient’s responses to the relevant items yields scores for each field and a total score that can be standardized on a scale from 0 to 100, with higher scores indicating higher patient satisfaction (Cronbach’s α ranging from 0.72 to 0.93).

#### WP3: quality, coordination and access to innovation

Quality will be evaluated based on the visits and treatment during a given period, whether the setting applies a multidisciplinary consultative framework, the management by nurse coordinators, the use of a personalized care plan, and the delivery of information documents related to treatments and supportive care.

Coordination and collaboration among structures and caregivers will be evaluated on several levels. The hospital care provider questionnaire addresses the practices of physicians who are in contact with the patient and the sharing of tools with outpatient caregivers. The referring physician questionnaire describes the role of the referring physician and his interaction with hospital caregivers. The center questionnaire addresses the hospital’s organization and activity and their labor and logistics expenses. An electronic case report form collects medical data and inpatient consumptions.

Several levels of collaboration will be assessed: the organization among different types of professionals within the same hospital, the collaboration among structures, and the individual collaborations among physicians within and outside the hospital.

Outpatient surgery, home hospitalization and one-stop breast clinic visits will be recorded to assess access to innovation.

### Statistical analysis

The statistical analysis will be conducted in three steps. First, a descriptive analysis will be conducted on the population studied and on the care pathways.

Second, homogeneous groups of patients will be established based on the patients’ individual medical information, such as age, surgical management procedures, and adjuvant therapy (radiotherapy and chemotherapy). For a given homogeneous group of patients, care pathways and endpoints will be compared. The endpoints are the costs of care pathways, patient satisfaction, work reintegration, readmissions and time lapses between care stages. The variability of these endpoints will be studied in light of the patients’ socio-demographic characteristics and geographical situation and the structure of care. The third part of the analysis will consist of a descriptive approach, regardless of the group to which the patient belongs. A multiple correspondence analysis will be conducted with care resource use and socio-demographic and medical characteristics as active variables. The variables that constitute the endpoints will be projected onto a space defined by appropriate axes.

## Discussion

In the United States, the diversity of care pathways is limited by managed care plans. Coverage restrictions encourage patients to follow a certain care pathway within a network of health care providers that contract with insurers. Historically, the French health care system is characterized by the founding principle of liberty: liberty both for physicians to practice and for patients to use health care services. Currently, to control growing health care expenses, a number of questions and considerable debate has focused on the organization of care pathways. However, it appears that there is no general or unique scope for these pathways and no established monetary valuation of the cost pathways for any disease. The French National Cancer Institute has specifically called for proposals on economic assessments of care pathways since 2013. The OPTISOINS01 study was the only project that was positively perceived, and it was selected for a national grant in 2013.

### The spatial and temporal scope of the care pathway still needs to be defined

To our knowledge, no term that describes the whole care process has been found in the literature. Authors often use the term “care pathway” without defining it specifically [13] or confuse the concept and the tool designed for its practical application (e.g., the European pathway association). The term “care pathway” is commonly used as a synonym for “clinical pathway”, “critical pathway”, “integrated care pathway” or “care map” to name the tool used to standardize care for a homogeneous group of patients. It lists in advance the acts to be performed by a single multidisciplinary team to meet established guidelines at a local level.

We chose to use the term “care pathway” to refer to the concept behind this quality improvement tool. As we use it, “care pathway” indicates the effective succession of care, structures and providers throughout the illness for each patient. The care pathways in our study are not planned but are observed as they happen. The succession of actors from several structures over a given period of time implies the temporal and spatial aspects of the care pathway. Because the notion of care pathway is global and includes the entire care process, the time frame and the spatial frame must be defined.

Several empirical studies have been published on the BC clinical pathway. Studies often compare patients’ situations before and after the clinical pathway implementation using selected indicators in the care process [14], and they sometimes compare costs [15, 16].

Some studies evaluate the 1-year costs of BC but do not give details about the care services that compose the care pathway [17] or assess only hospital-specific cost items, excluding the social and ambulatory care sectors [17, 18]. A model of the care pathway for new, non-metastatic BC has been proposed; it describes the temporal sequence of hospital care, but its costs were not assessed [19]. One published study evaluating BC pathways has been found in the literature. The authors assessed the 1-year care pathway of patients who underwent breast cancer surgery in 2009 in France. They proposed a method for grouping care pathways over a sequence of hospitalizations using claim data [18]. The pathway studied included only the hospital portion of the BC care at 1 year and did not refer to a specific health territory.

### The assessment of care pathways encourages the implementation of new payment models

One major challenge associated with the optimization of a cancer care pathway is the implementation of new prospective payment methods, such as patient-based bundled payments.

Hospitals are usually funded by a prospective payment system based on diagnosis-related groups (DRGs). Patient assignments to DRGs are based on their primary diagnosis. This classification system was adapted from the US Healthcare Financing Group classification. The DRGs’ prices (tariffs) are established annually at a national level based on average costs. The financing of health care services implies the involvement of an individual producer, such as the hospital, the laboratory, the self-employed physician or the radiology center for example. The prospective payment system encourages the segmentation and multiplication of examinations or visits.

New payment models aim to encourage the efficiency of care pathway. In the United States, bundled payment, also known as episode-based payment, has been proposed as a strategy for reducing health care costs during health care reform debates, especially during the Obama administration. This type of payment is currently being implemented for surgery cases [7]. It replaces payments for each provider during a health care episode. This payment approach offers a single or package payment and implies that initial negotiation among providers and contract with the payers (health insurance) has occurred. Using an overall approach, the payers assess the resources consumed during each care episode and valorize them according to the average cost linked to best practices. This method takes into account the dispersion of patient outliers, introducing variability into the average management pathway and limiting losses through “stop loss” and other contracts that limit the financial losses for each professional. Our study will provide confidence intervals for bundled payments according to practice settings and regional parameters.

Bundled payment is expected to increase the efficiency and quality of health care because under that model, the financial risks are held by the health care providers. It aims to promote sharing and coordination among health professionals and to encourage a patient-focused care pathway approach in accordance with clinical practice recommendations. It can also provide transparency for consumers by fixing pricing and by publishing cost and outcomes data. Some European countries have introduced episode-based payment: Portugal did so in 2008 for dialysis, and the Netherlands did so in 2010 for type two diabetes, chronic obstructive pulmonary disease (COPD) and vascular risk management [20, 21]. In France, several ongoing pilot studies are focusing on bundled payment for outpatient surgery [22], chronic renal insufficiency and radiation treatment for cancer.

The assessment of care pathways encourages the implementation of new payment models. Our approach could help health care professionals and policy makers establish other cost-of-illness studies and plan resource allocation on a patient basis rather than on a visit basis.
